# Editorial: Diseases in the COVID-19 epidemic

**DOI:** 10.3389/fendo.2023.1138797

**Published:** 2023-01-24

**Authors:** Nicola Veronese, Shaun Sabico

**Affiliations:** ^1^ Geriatrics Section, Department of Internal Medicine, University of Palermo, Palermo, Italy; ^2^ Chair for Biomarkers of Chronic Diseases, Biochemistry Department, College of Science, King Saud University, Riyadh, Saudi Arabia

**Keywords:** COVID - 19, musculo skeletal, diabetes, thyroid, risk

It’s been 3 years since the World Health Organization (WHO) announced a previously mysterious pneumonia-related outbreak in Wuhan, China, the epicentre of severe acute respiratory syndrome coronavirus-2 (SARS-CoV-2) that would ultimately lead to the coronavirus disease-19 (Covid-19) pandemic. As of January 5, 2023, Covid-19 has been responsible for a roughly estimated 7.5 million deaths and more than 662 million cases globally (1). Within that small time frame several variants emerged, as expected with viruses’ evolution over time. More importantly however and independent of the infection itself, was the high vulnerability of several populations for severe manifestations of Covid-19, also known as those with “pre-existing conditions”. This Research Topic entitled “*Diseases in the Covid-19 pandemic*”, is one of the special Research Topics of Frontiers in Endocrinology focusing on Covid-19 with the aim of gathering state-of-the-art studies relevant to Covid-19 as an exacerbating risk factor in several modern diseases affecting musculoskeletal health as well as endocrine disorders. The present Research Topic was able to compile 3 original studies involving children and adults and one meta-analysis, done in different geographical settings, covering a wide range of diseases and life-style factors affected by the pandemic.

In the observational study of Bevilacqua et al., they investigated the impact of Covid-19 on factors relevant for musculoskeletal health in 2 cohorts involving community-dwelling elderly of Caucasian origin. The smaller and older cohort (N=125, median age 84.3 years) was taken from the Hertfordshire Cohort Study (HCS) while the bigger and younger cohort (N=2469, median age 65.7 years) was taken from the Health and Employment After Fifty (HEAF) Study. In both cohorts, negative lifestyle changes during the first wave of pandemic was substantial and was most evident in physical activity, where 47% of HCS respondents and 44% of HEAF participants reported being less physically active during lockdown. Unhealthy changes in dietary and alcohol intake habits were found to be more common among younger participants and women, indicating that some harmful lifestyle modifications were age- and sex-specific.

In a retrospective study by Alshukairi et al. among Middle-Eastern children (2022), 146 children and adolescents (mean age 12.9 years) with osteogenesis imperfecta (OI) were investigated for Covid-19 outcomes. Among the participants, 83 (67%) were eligible for vaccine, of whom 16 were unvaccinated at the time of the study. Only 12 patients (8%) had confirmed Covid-19 infection, all of whom were mild cases and recovered fully with no hospitalization required, suggesting that OI may not be as impactful as other genetic disorders in terms of Covid-19 severity.

The third study in this Research Topic was large-scale nationwide investigation of Shestakova et al. conducted in the Russian Federation which involved 235, 248 patients with known diabetes mellitus (DM) (Type 1 N = 11, 058; Type 2 N = 224, 190). Case fatality rate (CFR) was elevated in both DM types (8.1% in T1DM and 15.3% in T2DM) and was both significantly influenced by the male sex [odds ratio, OR 1.25 (95% Confidence Interval, CI, 1.09-1.44) in T1DM and OR 1.18 (95% CI 1.15-1.21) in T2DM] elderly age (>65 years) [OR 4.44 (95% CI 3.75-5.24) in T1DM and OR 3.18 (95% CI 3.09-3.26) in T2DM] and longer duration of DM (≥ 10 years) [OR 2.46 (95% CI 2.06-2.95) in T1DM and OR 2.11 (95% CI 2.06-2.16) in T2DM]. Interestingly, non-vaccination status conferred protection in terms of lower CFR in both T1DM [OR 0.07 (95% CI 0.03-0.20)] and T2DM [OR 0.19 (95% CI 0.17-0.22)].

The last study was a meta-analysis conducted by Tian et al. involving 53 out of 702 articles that covered the association of Covid-19 severity and its association with thyroid diseases. All studies included had greater than 5 scores, indicating high quality assessment with no publication bias based on funnel plot analysis and no significant heterogeneity. Twenty-two out of the 53 reported severity status of Covid-19 and had a pooled effect size of 2.62 (95% CI 1.96-3.49; p<0.001) suggesting that those with thyroid diseases were significantly more likely to progress to severe Covid-19, particularly those with hypothyroidism [fixed effects OR 3.72 (95% CI 1.62-8.58; p = 0.002) and low T3 [OR 5.86 (95% CI 2.79 – 12.33; p <0.00001).

Cumulatively, the studies published under this Research Topic overwhelmingly confirm that certain pre-existing conditions such as thyroid diseases and DM negatively affect Covid-19 severity and outcomes, while genetic disorders manifesting in paediatric populations have little to no effect in Covid-19 severity. Factors such as decreased physical activity, especially among the elderly during lockdown, may be harmful not only to musculoskeletal health but also to Covid-19 susceptibility. Given that Covid-19 is likely to stay and be a part of modern human existence, it is worth noting that even in the absence of vaccines, individuals having these pre-existing conditions need to be constantly made aware of their vulnerability until such conditions are treated or at least, stabilized. Behaviours deemed favourable for Covid-19 infection, and weaker immune system in general, such as sedentary lifestyle and unhealthy dietary changes, should also be discouraged through persistent public health campaigns that encourage healthier lifestyles and better dietary choices. The Covid-19 pandemic may appear to be waning, but given the innate ability of the virus to evolve to different variants of varying virulence, it is important to constantly remind the general public that while modern tragedy is not yet over, the knowledge that the scientific community gathered to understand this disease is more than enough arsenal to survive this pandemic. All it takes is to translate this knowledge into practice.

## Author contributions

NV and SS have both made substantial, direct and intellectual contribution to the work and approved it for publication.
